# Potential reverse spillover of infectious bursal disease virus at the interface of commercial poultry and wild birds

**DOI:** 10.1007/s11262-020-01793-x

**Published:** 2020-09-24

**Authors:** Rania F. El Naggar, Mohammed A. Rohaim, Muhammad Munir

**Affiliations:** 1 Department of Virology, Faculty of Veterinary Medicine, University of Sadat City, Sadat, 32897 Egypt; 2 Department of Virology, Faculty of Veterinary Medicine, Cairo University, Giza, 12211 Egypt; 3 Division of Biomedical and Life Science, Lancaster University, Lancaster, Lancashire, LA1 4YG UK

**Keywords:** Viruses, Spill over, Evolution, Poultry

## Abstract

Recently, multiple spillover events between domesticated poultry and wild birds have been reported for several avian viruses. This phenomenon highlights the importance of the livestock-wildlife interface in the possible emergence of novel viruses. The aim of the current study was to investigate the potential spillover and epidemiological links of infectious bursal disease virus (IBDV) between wild birds and domestic poultry. To this end, twenty-eight cloacal swabs were collected from four species of free-living Egyptian wild birds (i.e. mallard duck, bean goose, white-fronted goose and black-billed magpie). Genetic and phylogenetic analysis of three positive isolates revealed that the IBDV/USC-1/2019 strain clustered with previously reported very virulent IBDV (vvIBDV) Egyptian isolates. Interestingly, two other wild bird-origin isolates (i.e. IBDV/USC-2/2019 and IBDV/USC-3/2019) grouped with a vaccine strain that is being used in commercial poultry. In conclusion, our results revealed the molecular detection of vaccine and vvIBDV-like strains in Egyptian wild birds and highlighted the potential role of wild birds in IBDV epidemiology in disease-endemic regions.

## Introduction

Infectious bursal disease (IBD) is an acute and highly contagious disease of chicks, and the clinical impact of IBD is mainly attributed to its severe immunosuppression especially in young chickens. The IBDV infection particularly targets and annihilates the precursors of antibody-producing B cells within the bursa of Fabricius (BF) [1]. Importantly, the damages to the BF are permanent, resulting in vaccination failure and expanded defencelessness to other diseases [2].

The IBDV is non-enveloped, icosahedral in shape, and carry double-stranded RNA genome within the genus *Avibirnavirus* of family *Birnaviridae* [3]. The IBDV is composed of segment A (~ 3.17 kb in length) and B (~ 2.8 kb in length) [3]. The segment A is comprised of two partially overlapping open reading frames (ORFs). The non-structural viral protein 5 (VP5) encoded by the first ORF, whereas the second ORF encodes a polyprotein. This polyprotein is eventually cleaved into two structural proteins known as VP2 and VP3, and a serine protease called VP4 [4–6]. The VP2 is the main structural protein and carries all the neutralizing epitopes, involved in virulence, cell tropism, and antigenic variation [7–9]. The RNA-dependent RNA polymerase (VP1) is encoded by Segment B [10], which plays critical functions in viral replication [11].

Out of two IBDV serotypes (i.e. I and II), only serotype I strains of IBDV are virulent in chickens. These strains are grouped into four characteristic pathotypes including classical, attenuated, antigenic variant, and very virulent strains [12–14]. Nearly 60–76% of IBDV isolates across four continents can be grouped as very virulent based on the global molecular epidemiological investigations [15]. Since the first report of the very virulent IBDV (vvIBDV) in the USA in 1957 [12, 16], the disease has been spreading worldwide [15] including Egypt [17–19] and has undergone a complex evolution. In Egypt, the vvIBDVs were first reported in 1989 [19]. To contain the infection, live-attenuated, intermediate plus, and classical strain-based vaccines are currently being used in the Egyptian poultry industry [20]. Despite mass vaccination regimes, Egypt is experiencing repeated IBDV outbreaks with high mortality rates since last two decades [17–19].

A relatively recent area of research at livestock-species interface is the spillover of viruses from the fared poultry into wild birds that can risk the wild birds' welfare. In commercial poultry farms, vaccination may have a significant effect on virus evolution [20] and possible spread to wild birds in vicinity [21, 22]. Several spillover events of vaccine viruses from domestic poultry to wild birds have been reported such as Newcastle disease virus and infectious bronchitis virus [21, 22]. Owing to high demands for free-range and backyard poultry production, the direct interaction between wild birds and farmed poultry is increasing [23]. Furthermore, massive size of the industrialized poultry production may risk the environment contamination with infectious materials through activities such as reuse of poultry litters [23].

This study was designed to investigate the potential spillover of infectious bursal disease virus (IBDV) between wild birds and domestic poultry. A total of 28 cloacal swabs were collected from Egyptian free-living wild birds during 2019, and genetics and transmission risks were assessed for the IBDV in Egypt.

## Materials and methods

### Samples collection, virus isolation, and genetic characterization

Twenty-eight cloacal swabs were collected from three Egyptian provinces (Monofiya, Qaulubia and Sharkia) during 2019, which were considered wild birds-dense and IBDV-endemic areas in the Nile Delta region (Table 1). The Nile Delta of Northern Egypt is a crucial stopover for millions of birds migrating between the Palearctic and Afrotropical regions annually, and considered one of the most important migration routes for wild birds [24, 25].

**Table 1:** Overview of wild bird samples involved in the study, and the prevalence of IBDVs in different species

Order	Family	Genus	Species	Region/Governorate	Sampled (n)	Positive (n)
Anseriformes	Anatidae	*Anas*	*A. crecca* (Green- winged teal)	Monofiya,	3	0
				Qaulubia	2	0
				Sharkia	3	1
			*A. platyrhynchos* (Mallard)	Monofiya,	2	0
				Qaulubia	3	0
				Sharkia	2	0
Pelecaniformes	Ardeidae	*Bubulcus*	*B. ibis* (Cattle egret)	Monofiya,	3	1
				Qaulubia	2	1
				Sharkia	1	0
Galliformes	Phasianidae	Coturnix	*C. coturnix* (Common quail)	Monofiya,	2	0
				Qaulubia	3	0
				Sharkia	2	0

n means: number

Capturing and sampling from live wild birds were carried out in accordance with all relevant guidelines, regulations and animal ethics permits issued by the Faculty of Veterinary Medicine, University of Sadat City, Egypt. The cloacal swabs were collected from each bird individually and placed in 1.5 ml of phosphate buffer saline (PBS) supplemented with 2000 unit/ml Penicillin G, 200 mg/ml Gentamicin, and 4 mg/ml Amphotericin B. The swab fluids were clarified by centrifugation at 1500 rpm for 10 min, and the supernatant was used for RNA extraction using TRIzol™ reagent as per manufacturer’s instructions. Using RT-PCR assays, the extracted RNA were screened for IBDV using a primer pair that amplifies a 743 bp region of VP2 gene, the forward primer was 5′-GCC CAG AGT CTA CAC CAT-3′ and the reverse primer was 5′-CCC GGA TTA TGT CTT TGA-3′ [26].

The RT-PCR-positive samples (*n* = 3) were inoculated on the chorioallantonic membrane (CAM) of specific pathogen free (SPF) embryonated chicken eggs following the standard procedures [27]. Five days post-inoculation, all embryos died. CAMs were harvested from dead embryos and screened by qRT-PCR for IBDV. The RNA was extracted from positive CAMs using TRIzol™ reagent as per manufacturer’s instructions (Invitrogen, USA). The extracted RNA treated with dimethylsulphoxide (DMSO) for 5 min at 98 °C and then snap chilled [27]. The synthesis of cDNA from the DMSO-treated RNA was performed using SuperScript™ IV Reverse Transcriptase (Thermo Scientific, USA) as per the manufacturer’s instruction. Polymerase chain reaction (PCR) was carried out using High Fidelity Q5 polymerase (NEB, UK), according to manufacturer’s instructions for the amplification of full length VP2 gene using the following primers; IBDVP2F-5′-ATG ACA AAC CTG CAA GAT CAA ACC CAA C-3′ and IBDVP2R-5′-TTA TGT CTT TGA AGC CAA ATG CTC CTG C-3′. These primers flank the conserved regions of VP2 ORF among IBDV serotype I strains. Briefly, a total of 50 μl reaction mixture contain 2 μl of cDNA, 10 μl of 5X Q5 Reaction Buffer, 10 ul of 5X Q5 High GC Enhancer 2.5 μl primer IBDVP2F, 2.5 μl primer IBDVP2R, 2 μl dNTPs mix, 0.5 μl of Q5 High-Fidelity DNA Polymerase and 20.5 μl nuclease free water. The PCR cycling protocol was as follows: 98 °C for 3 min followed by 40 three-step cycles of 98 °C for 30 s, 68 °C for 45 s and 72 °C for 2 min; then 72 °C for 10 min. The PCR products were analysed on electrophoreses using a 1% agarose gel containing ethidium bromide and were visualized under UV illumination. The QIAquick Gel Extraction Kit (Qiagen, Germany) was used to purify the PCR products. These products were sequenced bi-directionally with both sense (IBDVP2F) and antisense (IBDVP2R) primers that were used in the PCR amplification. The sequencing was performed utilizing BigDye terminator v3.1 cycle sequencing kit in an ABI 3100 genetic analyser (Applied Biosystems Foster City, California, USA).

### Sequence analysis, phylogeny, and selective pressure analysis

Nucleotide sequences were aligned with ClustalW [28] and analysed using the BioEdit 5.0 package [29]. The obtained nucleotide sequences were submitted to GenBank and are available under the accession numbers; MT304668-MT304670. Sequence Demarcation Tool (SDT) was used to display the nucleotide pairwise identity scores through a color-coded matrix [30]. Phylogenetic analyses were carried out using general time-reversible (GTR) model [31], which was selected using jModelTest [32], and maximum-likelihood trees were constructed using RaxML version 8.2.11 [33] with 1000 bootstrap replicates.

The VP2 gene-specific estimates of dN/dS were predicted using the Synonymous-Non-Synonymous Analysis Program (SNAP) [34]. The number of potential synonymous and non-synonymous changes were counted as well as the number of actual synonymous and non-synonymous changes in codon between each pair. The dN/dS ratio was calculated by comparing the proportion of observed non-synonymous substitutions over the proportion of observed synonymous substitutions. These were then adjusted for multiple hits using the Jukes–Cantor correction [34].

## Results and discussion

Understanding the epidemiology of vvIBDV is important to underpin the viral evolution, virus spread and up-to-date field status for effective control strategies. Previous studies have reported a widespread usage of live vaccines help in the spread of IBDVs with emergence of vaccine escape mutant strains [35–38]. Based on serological evidences of IBDV serotype I in wild birds, it has been suggested that wild birds may be critical player in the epidemiology of IBDV and may act as reservoir for the IBDV [39–43].

Usage of live vaccines is blamed to be responsible for spillover of viral vaccines from poultry into wild birds [22, 23] The safety of attenuated IBDV vaccines that are commonly used in the Egyptian poultry sectors might be examined systematically within the commercial avian species but not in wild birds that might be susceptible to infection [24]. In spite of restricted epidemiological studies for viruses in wild birds, spilling over of poultry vaccines has been documented in wild birds [24]. Despite the direct impacts of the attenuated viral vaccines on wild birds, the potential for these vaccines to develop significant levels of pathogenicity in wild birds is a major challenge [44]. These findings highlight the potential roles of wild birds in the spread of IBDV. In the current study, twenty-eight samples were collected from randomly selected wild birds from three Egyptian Governorates These samples were individually screened for IBDV by the RT-PCR targeting the VP2 gene. Three samples (3 out of 28) were identified positive among the tested cloacal samples (Table 1). The sampled wild birds were classified into four different families; Anatidae (*A. crecca* species, *n* = 8 and *A. platyrhynchos* species, *n* = 7), Ardeidae (*B. ibis* species, *n* = 6) and Phasianidae (*C. coturnix* species, *n *= 7) based on their taxonomy (Table 1). The vvIBDV isolate Egypt-USC-IBD-1-2019 was collected from *B. ibis* species of Qaulubia Governorate while the IBDV vaccine-like strains Egypt-USC-IBD-2-2019 and IBDV isolate Egypt-USC-IBD-3-2019 were isolated from *A. crecca* species*,* Sharkia Governorate and *B. ibis* species, Monofiya Governorate, respectively. Identification of these IBDV in birds from the Nile Delta of Northern Egypt is of particular concern. The Nile Delta is historically a crucial stopover for millions of birds. These birds migrate between the Palearctic and Afrotropical regions every year. Therefore, the Nile Delta is considered one of the most important migration routes for wild birds [24, 25]. Circulation of IBDV in these wild birds could pose a risk of infection to other birds migrating through multiple routes.

**Fig. 1:**
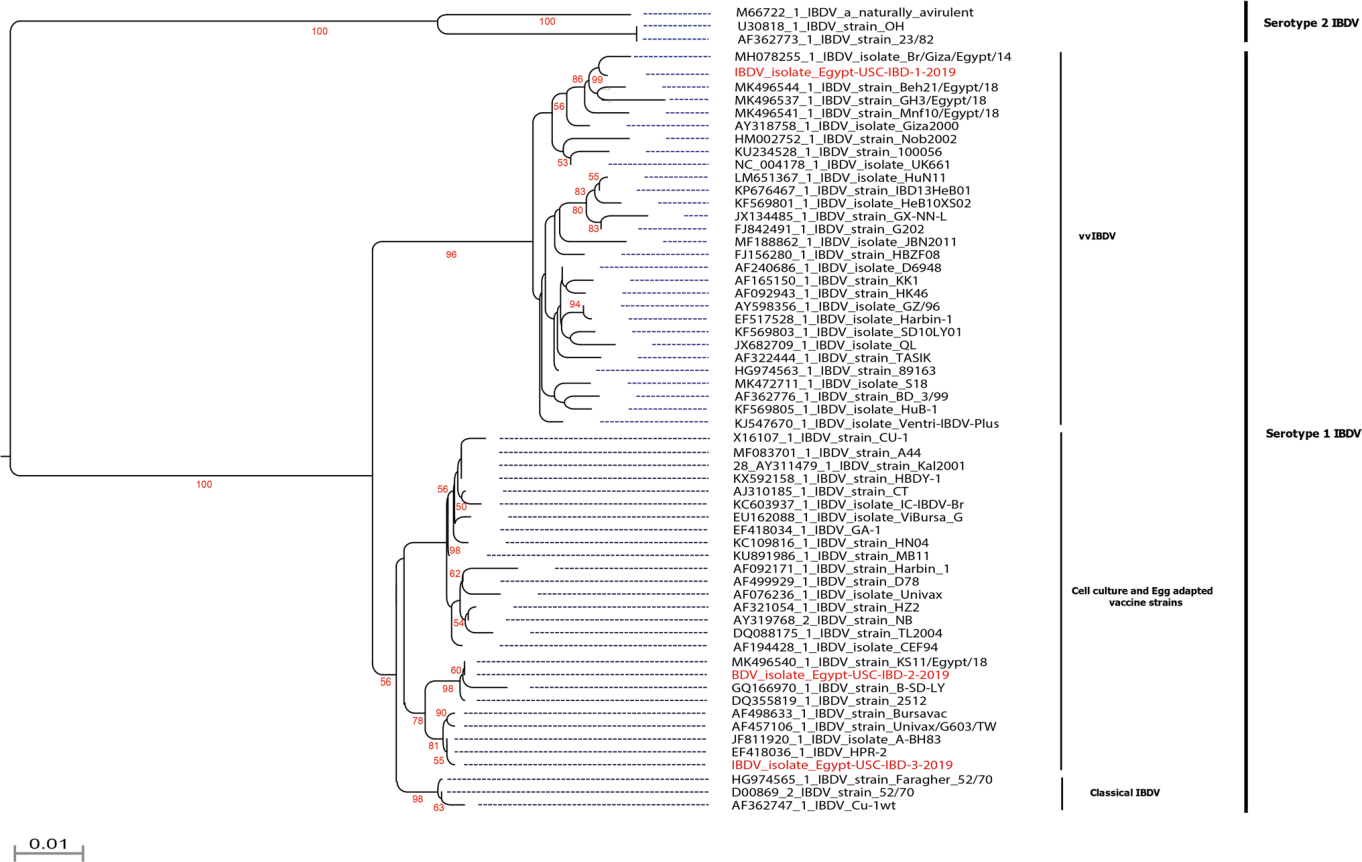
Phylogenetic analysis of studied isolates and their clustering patterns with representative IBDVs. Full length VP2 gene based phylogenetic analysis of three wild-bird origin IBDV isolates with representative strains of currently circulating IBDVs in Egypt. One of the reported isolates clustered within vvIBDVs with close relationship with the previously characterized strains from commercial poultry while the other one clustered vaccine strains. The reported isolated marked with red colour

The phylogenetic analysis based on VP2 sequences revealed that IBDV isolate Egypt-USC-IBD-1-2019 clustered with vvIBDV (Fig. 1) whereas two other isolates (IBDV isolate Egypt-USC-IBD-2-2019 and IBDV isolate Egypt-USC-IBD-3-2019) clustered with cell-culture adapted IBDV vaccine strains (Fig. 1). The highly variable domain of VP2 protein carries the antigenic region which is accountable for neutralizing antibody as well as virulence [9]. Genetic analysis of the highly variable domain of VP2 may help to identify the genetic relationship among IBDV strains [9]. Previous studies have demonstrated that there are two major and three minor hydrophilic regions within the VP2 [45]. The major hydrophilic regions are represented by peak A (aa 212–224) and B (aa 314–324) while the three minor hydrophilic regions ranged from aa 248–252, 279–290 and 299–305 [45]. Likewise, there is a serine-rich heptapeptide _326_SWSASGS_332_ sequence close to the second major hydrophilic region, was found in virulent strains and it might be the virulence marker for IBDV [45] which is detected in the isolated vvIBDV characterized in this study. Previous reports demonstrated the structural conformation of the major hydrophilic peaks A and B as critical in determining the IBDV antigenicity. Overall, finding revealed high selection pressures in peak A and B, and highlight key amino acids that can play critical roles in preserving the structural confirmation of the VP2 protein and decide the magnitude of virulence, pathogenicity and characterization of IBV.

**Fig. 2:**
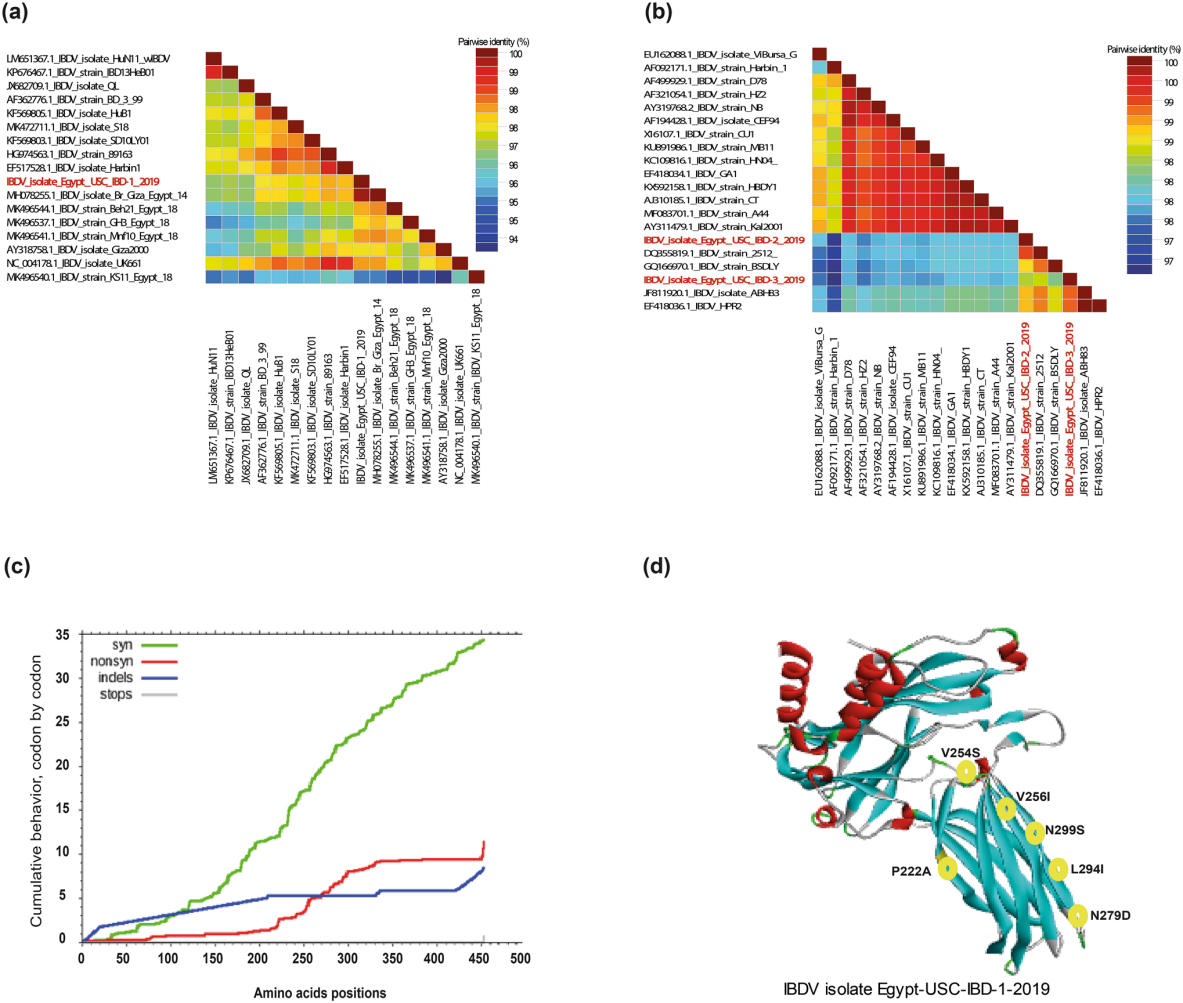
Pairwise identity, localization of specific mutations in the VP2 protein of the newly identified vvIBDV strain and IBDVs selective pressure. The pairwise identities plot of VP2 gene for **a** Egypt-USC-IBD-1-2019 compared to vvIBDVs and **b** Egypt-USC-IBD-2-2019 and Egypt-USC-IBD-3-2019 compared to IBDV vaccine-like strains aligned by ClustalW and displayed by Sequence Demarcation Tool (SDT) software. **c** Cumulative behaviour of the average synonymous and non-synonymous substitutions moving codon by codon across VP2 gene. **d** 3D structure template for IBDV isolate IBDV/USC-3/2019 showed the localization of specific mutations in the VP2 protein for IBDV isolate IBDV/USC-1/2019. The 3D was visualized by PyMOL software

Our analysis of VP2 gene sequences indicated that wild bird-origin IBDV isolates carried high similarity with vvIBDV (Fig. 2a) and vaccine strains (Fig. 2b) previously reported from domestic chickens in Egypt [17]. Moreover, the presence of IBDV in the cloacal swabs of the wild birds suggested that these birds can shed the virus without developing disease, which may have implications in the IBDV epidemiology. These data suggest an epidemiological link between domestic chickens and wild birds in the epidemiology of IBDVs. Previous studies have demonstrated the serological presence of IBDV in multiple wild bird species [39–43]. Since serotype I of IBDV is known to be a pathogenic in avian species other than chicken, it become clear that IBDV didn't assume a significant role in the bird’s deaths [46]. Our results revealed that such isolates are most likely spilt over from previous outbreaks in vaccinated poultry and are carried by free-living wild birds, which may be playing a role in their dissemination. It has been well recognized the spillover of wild birds’ viruses to domesticated poultry causing disease and also in the other direction (from poultry to wild birds) [21, 22]. Previous studies have reported that passaging of NDV vaccine strains in wild bird species may provide selective pressures that could lead to antigenic variabilities or an increase in virulence [47–49]. The VP2 gene-specific estimates of dN/dS were predicted using SNAP and the number of potential synonymous and non-synonymous changes were counted. The sites under positive or negative selection were mapped and outlined in Fig. 2c.

Interestingly, the VP2 gene of Egypt-USC-IBD-1-2019 vvIBDV isolated from wild bird gained specific amino acid mutations (P222A, V256I, N279D, L294I, and N299S) (Fig. 2d), which are conserved among all Egyptian vvIBDV strains. However, a unique amino acid mutation (G254S) was observed in the studied isolates (Fig. 2d). These results suggested an existing close link between the IBDV epidemiology in both domesticated chickens and wild birds. The IBDV strains characterized from wild birds may be infectious and virulent in chickens and warrant future investigations. Although the number of samples analysed in this study were limited, it is plausible that the circulation of IBDVs among wild birds is much higher than previously thought. Continuous disease monitoring, surveillance, and subsequent complete viral genome characterization is advisable in case of spillover from wild birds to commercial poultry and/or reverse spillover from commercial poultry to wild birds.

Future investigations are warranted to underpin the proposed virulence markers as guidelines for the cataloguing of IBDV strains into diverse pathotypes. Additional animal trials of the currently used commercial inactivated IBDV vaccines are needed to confirm their effectiveness against field IBDV strains without the use of live IBDV vaccines. To further understand the transmissibility of the wild bird-origin IBDV strains, additional experiments such as assessment of the minimum infectious and lethal doses need to be performed. Thus, further research is needed to investigate the pathobiology of wild bird-origin IBDVs that might help to explore the pathobiology and immunosuppressive impacts of IBDV isolates and tracking their evolutionary changes to better assess the nature of recently circulating strains of IBDV.

## Data Availability

All sequence data are available in GenBank, and their accession numbers are MT304668-MT304670.
