# Correction to: Potential reverse spillover of infectious bursal disease virus at the interface of commercial poultry and wild birds

**DOI:** 10.1007/s11262-021-01824-1

**Published:** 2021-01-30

**Authors:** Rania F. El Naggar, Mohammed A. Rohaim, Muhammad Munir

**Affiliations:** 1 Department of Virology, Faculty of Veterinary Medicine, University of Sadat City, Sadat, 32897 Egypt; 2 Department of Virology, Faculty of Veterinary Medicine, Cairo University, Giza, 12211 Egypt; 3 Division of Biomedical and Life Science, Lancaster University, Lancaster, LA1 4YG Lancashire UK

## Correction to: Virus Genes (2020) 56:705–711 10.1007/s11262-020-01793-x

The original version of this article unfortunately contained an error in figure.

Figures 1 and 2 have interchanged their positions in the article. The correct Figs. 1 and 2 are presented here.

**Fig. 1:**
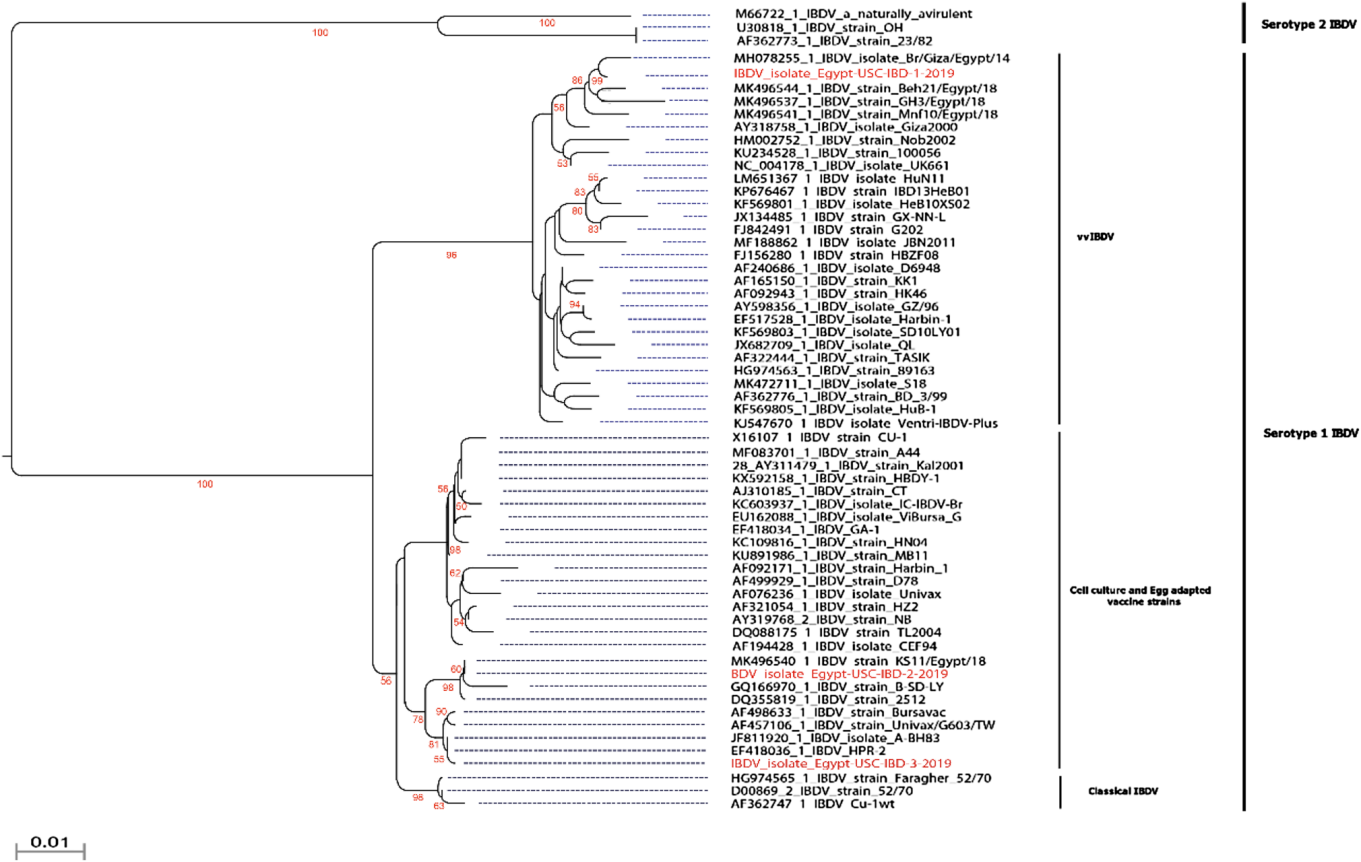
Phylogenetic analysis of studied isolates and their clustering patterns with representative IBDVs. Full length VP2 gene based phylogenetic analysis of three wild-bird origin IBDV isolates with representative strains of currently circulating IBDVs in Egypt. One of the reported isolates clustered within vvIBDVs with close relationship with the previously characterized strains from commercial poultry while the other one clustered vaccine strains. The reported isolated marked with red colour

**Fig. 2:**
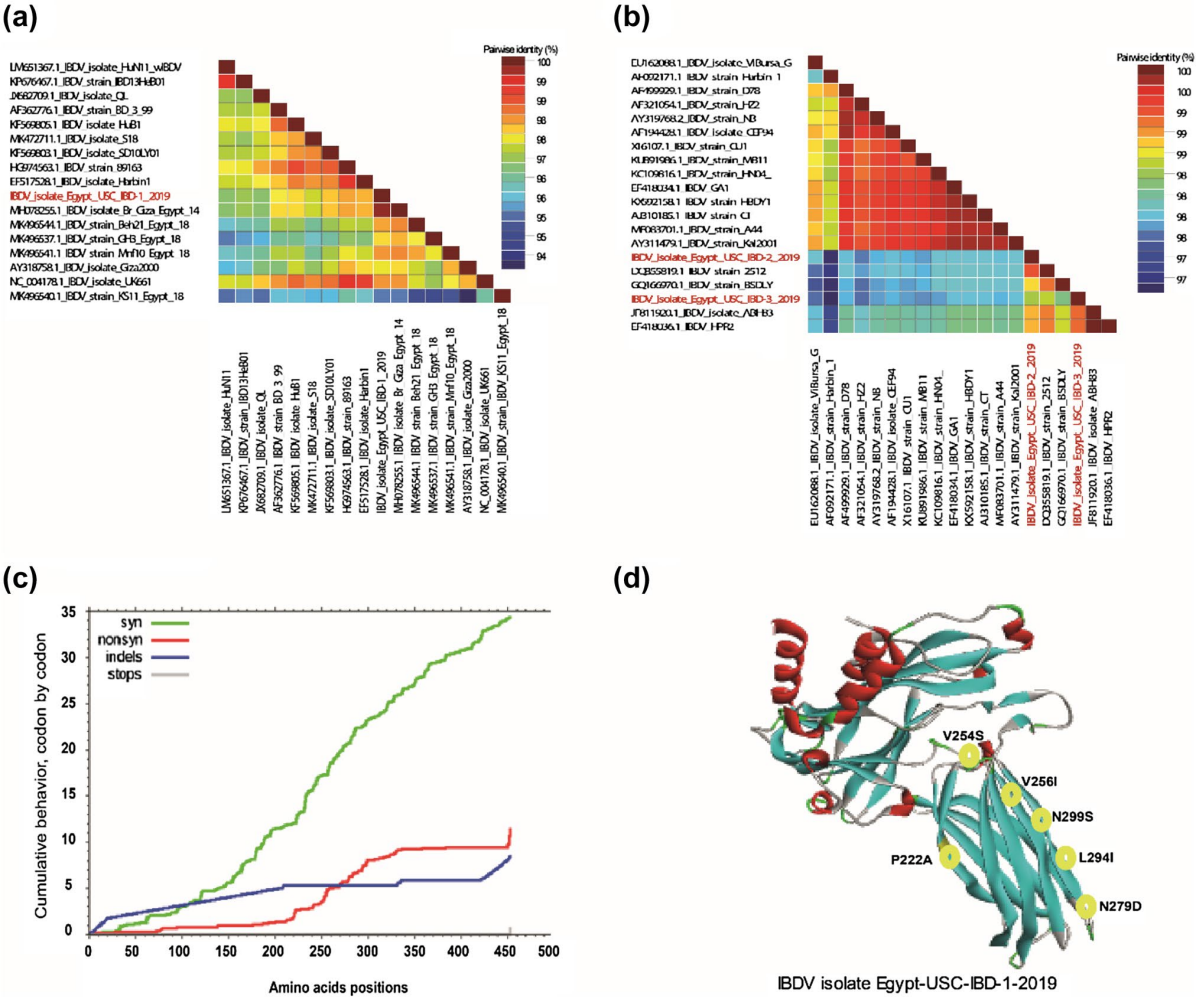
Pairwise identity, localization of specific mutations in the VP2 protein of the newly identified vvIBDV strain and IBDVs selective pressure. The pairwise identities plot of VP2 gene for **a** Egypt-USC-IBD-1-2019 compared to vvIBDVs and **b** Egypt-USC-IBD-2-2019 and Egypt-USC-IBD-3-2019 compared to IBDV vaccine-like strains aligned by ClustalW and displayed by Sequence Demarcation Tool (SDT) software. **c** Cumulative behaviour of the average synonymous and non-synonymous substitutions moving codon by codon across VP2 gene. **d** 3D structure template for IBDV isolate IBDV/USC-3/2019 showed the localization of specific mutations in the VP2 protein for IBDV isolate IBDV/USC-1/2019. The 3D was visualized by PyMOL software

The original article has been corrected.

